# Optimization of isocenter position for multiple targets with nonuniform‐margin expansion

**DOI:** 10.1002/acm2.13853

**Published:** 2022-11-21

**Authors:** Junjie Miao, Yingjie Xu, Jianrong Dai

**Affiliations:** ^1^ Department of Radiation Oncology National Cancer Center/National Clinical Research Center for Cancer/Cancer Hospital Chinese Academy of Medical Sciences and Peking Union Medical College Beijing China

**Keywords:** adaptive simulated annealing, nonuniform margin, setup uncertainty, single isocenter multiple targets

## Abstract

**Purpose:**

The single isocenter for multiple‐target (SIMT) technique has become a popular treatment technique for multiple brain metastases. We have implemented a method to obtain a nonuniform margin for SIMT technique. In this study, we further propose a method to determine the isocenter position so that the total expanded margin volume is minimal.

**Materials and method:**

Based on a statistical model, the relationship between nonuniform margin and the distance *d* (from isocenter to target point), setup uncertainties, and significance level was established. Due to the existence of rotational error, there is a nonlinear relationship between the margin volume and the isocenter position. Using numerical simulation, we study the relationship between optimal isocenter position and translational error, rotational error, and target size. In order to find the optimal isocenter position quickly, adaptive simulated annealing (ASA) algorithm was used. This method was implemented in the Pinnacle^3^ treatment planning system and compared with isocenter at center‐of‐geometric (COG), center‐of‐volume (COV), and center‐of‐surface (COS). Ten patients with multiple brain metastasis targets treated with the SIMT technique was selected for evaluation.

**Results:**

When the size of tumors is equal, the optimal isocenter obtained by ASA and numerical simulation coincides with COG, COV, and COS. When the size of tumors is different, the optimal isocenter is close to the large tumor. The position of COS point is closer to the optimal point than the COV point for nearly all cases. Moreover, in some cases the COS point can be approximately selected as the optimal point. The ASA algorithm can reduce the calculating time from several hours to tens of seconds for three or more tumors. Using multiple brain metastases targets, a series of volume difference and calculating time were obtained for various tumor number, tumor size, and separation distances. Compared with the margin volume with isocenter at COG, the margin volume for optimal point can be reduced by up to 27.7%.

**Conclusion:**

Optimal treatment isocenter selection of multiple targets with large differences could reduce the total margin volume. ASA algorithm can significantly improve the speed of finding the optimal isocenter. This method can be used for clinical isocenter selection and is useful for the protection of normal tissue nearby.

## 1 INTRODUCTION

It is estimated that 20%–40% of cancer patients will have brain metastases at some point.^1^ Malignant brain diseases cause a huge medical burden and require efficient and effective treatment. Stereotactic radiosurgery (SRS) for brain metastases can be performed by intensity modulated radiation therapy or volume‐modulated arc therapy with a C‐arm mounted linear accelerator.

In SRS, beams with steep dose gradients can deliver the radiation dose to the target while minimizing damage to surrounding healthy tissue. Historically, an isocenter was assigned to each target to reduce the uncertainty caused by setup errors. However, if multiple targets are treated for a patient, the treatment time may become unacceptable because the patient must be sequentially translated to each different isocenter for dose delivery. Usually the treatment time is about 20 min for one lesion and several hours for more than ten lesions.^2^

For busy clinical departments, shortening treatment time is desirable, which allows more patients to be treated in a fixed period of time or conversely the same number of patients can be treated in a shorter period of time. In order to shorten treatment time, several groups have studied the dosimetry and feasibility of using a single isocenter to treat multiple brain targets with SRS.^2^, ^3^, ^4^

For single‐isocenter multi‐target treatments, when the distance between the isocenter and the target is large, the small rotation error cannot be ignored. It was reported that when the rotation error (>1°) was introduced, the target coverage was significantly lower than expected.^5^ Chang developed a new margin recipe using statistical models that extra PTV margin required to maintain a given coverage probability for targets at known distances from the isocenter.^6^, ^7^

However, Chang's work did not provide a method for selecting isocenter locations to minimize the margin volume. Choosing the isocenter to minimize the nonclinical target volume is an important planning decision. It is because the volume of normal brain tissue receives 12 Gy (*V*_12 Gy_) is a known predictor of brain radiation necrosis.^8^ There are few studies on the selection of treatment centers in radiotherapy plans. In practice, the treatment center is often placed at the geometric center of all targets.

Recently, Slagowski introduced a method based on numerical simulation to select isocenter location of the treatment with two and three targets.^9^ However, there is a trend that patients with a higher number (more than five) of lesions are also treated with SRS.^10^, ^11^, ^12^ For these situations, the numerical simulation method needs to find the optimal isocenter in three‐dimensional space. Especially when the distance among the targets is large, the calculation will take long time. Cui et al. also introduced a method to select treatment isocenter location using stochastic optimization.^13^ However, those studies^9^, ^13^ can only be applied to uniform margin.

In order to avoid excessive compensation for the target points near isocenter, a nonuniform‐margin expansion was recently proposed.^14^ Compared with the uniform‐margin algorithm, the advantage of this method is that it will minimize the PTVs volume for given CTVs to obtain same significance level. In this method, the margin of each point on the target edge needs to be calculated separately. For nonuniform‐margin expansion, the calculating time using numerical simulation method would be unacceptable.

In this study, we propose a method to select the isocenter position for multiple targets that minimizes the total volume of expanded. This method is based on a statistical model considering both the conventional translational error and the additional rotational error. In order to find the optimal solution quickly, an adaptive simulated annealing (ASA) technique was used in this study. This method was implemented in the Pinnacle^3^ treatment planning system and compared with isocenter at the center‐of‐geometric (COG), center‐of‐volume (COV), and center‐of‐surface (COS).

## 2 MATERIALS AND METHODS

### 2.1 The statistical model

**FIGURE 1:**
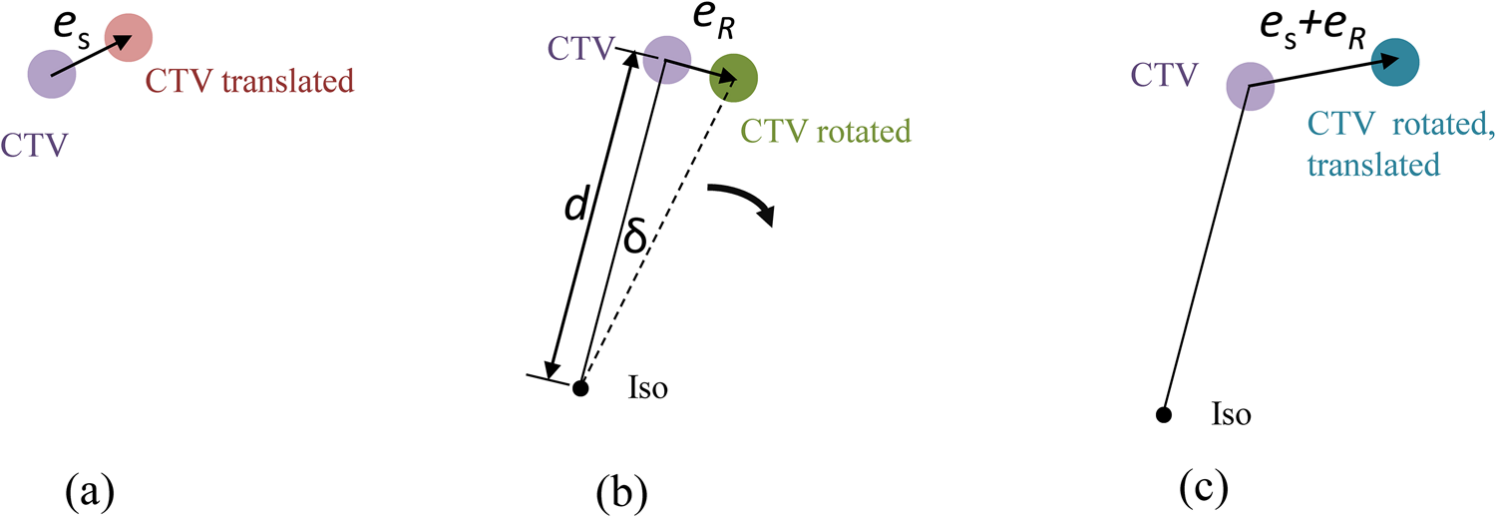
Illustration of (a) translational errors, (b) rotational errors, and (c) combined effect of the translational and rotational errors

Figure 1 illustrates the translational and rotational errors that introduce uncertainty to the CTV location. The translational error vector ***e***_S_ as shown in Figure 1a is a random vector with fixed amplitude and direction regardless the CTV location. In Figure 1b, the CTV rotates around the axis that is normal to the paper passing through the isocenter (ISO) for *δ* degrees. The rotational error ***e***_R_ is also a random vector. However, its amplitude is equal to sin*δ* × *d* ≈ *δd* (for small *δ*), where *d* is the distance between the isocenter and the CTV point. Moreover, its direction is not fixed but along the rotational direction. When considering the CTV to PTV expansion, the combined setup error ***e***_R_ + ***e***_S_ (rotated and translated) as shown in Figure 1c needs to be compensated.

**FIGURE 2:**
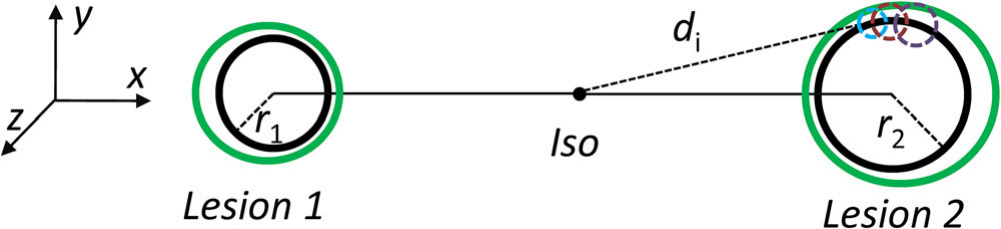
The influence of translational and rotational uncertainties on the margin of CTV. The offset caused by the rotation error depends on the distance from tumor to isocenter. The green structure in the figure represents PTV and the black one represents CTV.

Based on the published statistical model,^6^, ^7^ the translational setup error follows the three‐dimensional independent normal distribution with zero mean and a standard deviation (SD) of *σ*_s_ (in mm). It is assumed that the rotation happens randomly and follows a three‐dimensional independent normal distribution with a zero mean and an SD of *σ*_D_ (in degrees). Correspondingly, rotational setup error determined by rotation also follows a three‐dimensional independent normal distribution with a zero mean and an SD of $\sigma_{R}=0.816d\sigma_{D}\frac{\pi }{180}=0.01424d\sigma_{D}$ (in mm). The value of *σ*_R_ is proportional to *d* (the distance between the isocenter and the CTV point) and rotational uncertainty *σ*_D_. For a target, it can be thought as a combination of many small volume elements or target points. Based on the published method,^7^ the combined margin for the point *i* on CTV is

(1) $$\begin{array}{ccc} M_{i} & = & \chi_{\alpha }\sigma_{iE}=\chi_{\alpha }\sqrt{{\sigma_{iS}}^{2}+{\sigma_{iR}}^{2}}\\ & = & \chi_{\alpha }\sqrt{{\sigma_{S}}^{2}+{\left(0.01424d_{i}\sigma_{D}\right)}^{2}}=\sqrt{{M_{iS}}^{2}+{M_{iR}}^{2}}, \end{array}$$

where $\chi_{\alpha }^{2}$ is the critical value of chi‐squared distribution with three degrees of freedom for significance level *α*. In this study, $\chi_{\alpha }$ was set to 2.795 (corresponding to a significance factor of 0.95). $M_{iS}=\chi_{\alpha }\sigma_{iS}=\chi_{\alpha }\sigma_{S}=M_{S}$ is the required PTV margin for the translational error that is the same for all points of the target, and $M_{iR}=\chi_{\alpha }\sigma_{iR}$ is the required PTV margin for the rotational error. From Equation (1), it is clear that the combined PTV margin *M_i_* is related with translational margin *M*_S_, rotational uncertainty *σ*_D_, and the distance *d_i_* (between the isocenter and the CTV point *i*). The distance *d_i_* is

(2) $$d_{i}=\left|{\vec{s}}_{i}-{\vec{s}}_{0}\right|=\sqrt{{\left(x_{i}-x_{0}\right)}^{2}+{\left(y_{i}-y_{0}\right)}^{2}+{\left(z_{i}-z_{0}\right)}^{2}},$$

where (*x*_0_, *y*_0_, *z*_0_) is the coordinate value of the isocenter that is described by the vector ${\vec{s}}_{0}$. Moreover, (*x_i_*, *y_i_*, *z_i_*) is the coordinate value of the boundary point that is described by the vector ${\vec{s}}_{i}$. The influence of translational and rotational uncertainties on the margin of CTV is shown in Figure 2.

### 2.2 Objective function

Based on the above statistical model, the margin of each point could be obtained using Equation (1). The whole CTV–PTV margin is determined by all possible margins of the boundary points. The whole volume of CTV–PTV margin is related to the location of the isocenter. The margin volume can be expressed as

(3) $$V({\vec{s}}_{i},{\vec{s}}_{0})=V_{\mathrm{PTV}}-V_{\mathrm{CTV}}.$$

The vector ${\vec{s}}_{0}$ (*x*–*y*–*z* coordinates) that minimizes the sum of margin volume corresponds to the position of the optimal isocenter. Objective function is

(4) $${\hat{\vec{s}}}_{0}=\underset{{\vec{s}}_{0}}{\mathrm{argmin}}\;V({\vec{s}}_{i},{\vec{s}}_{0}).$$

### 2.3 Methods’ realization

The numerical simulation method is to calculate all the targets volume when the isocenter iterates over all possible points in three‐dimensional space defined by the center of each target. Then the point corresponding to the smallest margin volume is chosen as the optimal isocenter. When the number of tumors exceeds three, numerical simulation will search the optimal isocenter in three‐dimensional space. For nonuniform‐margin expansion, the time required for numerical simulation could be very long. Moreover, the time required for numerical calculation is clinically unacceptable.

In order to increase the speed of searching optimal isocenter, we used ASA method in this study. In the ASA method, the geometric center is used as the initial isocenter, and the solution space is the same as the numerical simulation method. ASA is an open source variant of simulated annealing that is used to search for isocenter location that optimizes Equation (4). ASA is considered a probabilistic optimization method with a statistical guarantee of global minimization. In ASA, the introduction of reannealing permits adaptively changing sensitivities in the multidimensional solution space, and its annealing schedule is faster than the Boltzmann annealing. ASA was developed by Ingber^15^ and has been previously used in radiation therapy.^16^, ^17^ The ASA program is written in standard C language. The source code and detailed description of ASA can be found on this website (www.ingber.com).

Our calculations were finished in personal office computer (CPU Intel Core i5‐4590 3.3 GHz, RAM 32 GB). The isocenter position, the margin volume, and calculation time of numerical simulation and ASA were recorded and compared. In order to facilitate the comparison, all the margin volume was normalized by the margin volume when isocenter is placed at COG.

### 2.4 Evaluation

The margin volume obtained by the ASA method was compared with that obtained by other isocenter settings for each combination of separation distance, CTV dimension, and setup uncertainty. The evaluated isocenters were placed at (i) the COG, (ii) the COV, (iii) the COS, (iv) the point of the numerical simulation optimal (NSO), and (v) the ASA optimal location that minimized the margin volume. For a given CTV indexed by *i* with its centroid's position described by coordinates (*x_i_*, *y_i_*, *z_i_*), the COG position is described by the following equation:

(5) $$\mathrm{COG}=\left(\frac{1}{n}\sum_{i=1}^{n}x_{i},\;\frac{1}{n}\sum_{i=1}^{n}y_{i},\;\frac{1}{n}\sum_{i=1}^{n}z_{i}\right).$$

The COV position is described by the following equation:

(6) $$\mathrm{COV}=\left(\frac{1}{V}\sum_{i=1}^{n}k_{i}x_{i},\;\frac{1}{V}\sum_{i=1}^{n}k_{i}y_{i},\;\frac{1}{V}\sum_{i=1}^{n}k_{i}z_{i}\right),$$

where *k_i_* is the volume of the target indexed by *i*,

(7) $$V=\sum_{i=1}^{n}k_{i}.$$

The COS position is described by the following equation:

(8) $$\mathrm{COS}=\left(\frac{1}{A}\sum_{i=1}^{n}a_{i}x_{i},\;\frac{1}{A}\sum_{i=1}^{n}a_{i}y_{i},\;\frac{1}{A}\sum_{i=1}^{n}a_{i}z_{i}\right),$$

where *a_i_* is the surface area of the target indexed by *i*,

(9) $$A=\sum_{i=1}^{n}a_{i}.$$

Based on this model, we selected ten patients with multiple brain metastasis targets treated with the single isocenter for multiple targets (SIMT) technique to test the impact of the isocenter selection method in clinical setting. The translation margin was set to 2 mm and rotational uncertainties were set to 1.0°.^18^, ^19^, ^20^, ^21^ The number of brain metastases in each plan ranges from two to six. The separation distance ranges from 28.5 to 135.5 mm. For each patient, the margin volume was computed and compared with a 95% probability of target coverage for all isocenters. We also compared the calculation time taken by the numerical simulation and the ASA method.

## 3 RESULTS

### 3.1 Two tumors

**FIGURE 3:**
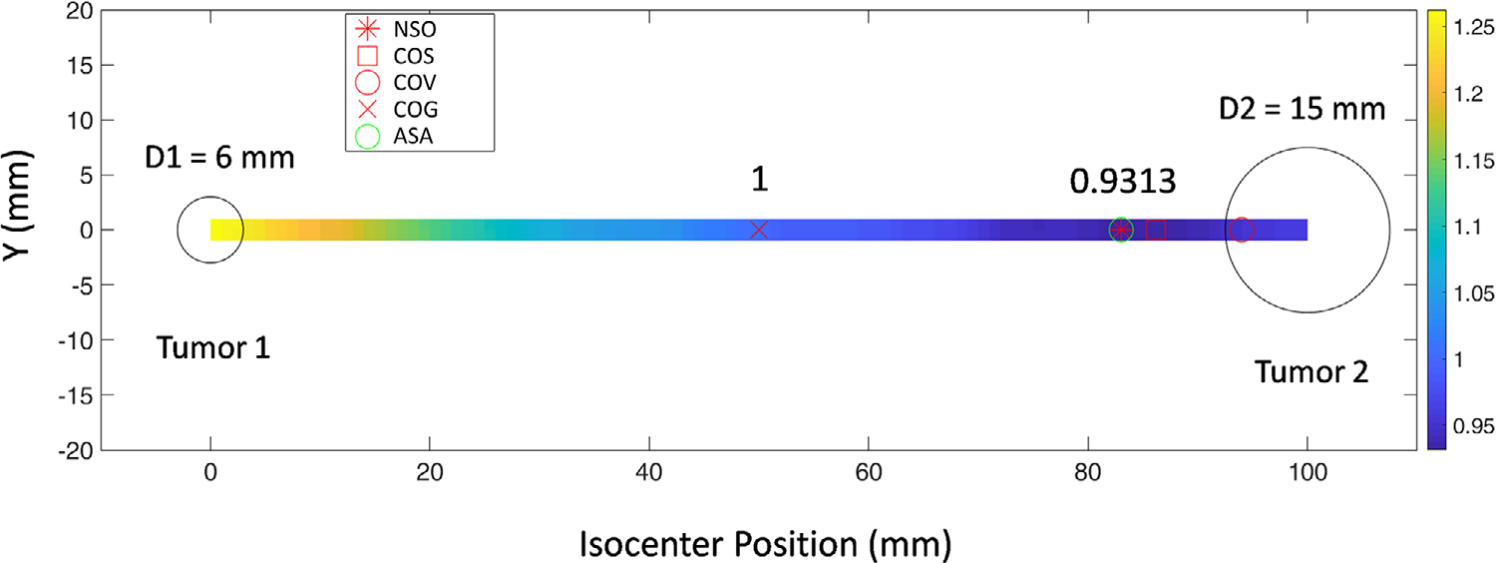
An example of normalized margin volume distribution is shown for *M*_S_ = 2 mm, rotational uncertainty is 0.5°. The distance between the two targets is 100 mm. In the heat map, the color represents relative margin volume when isocenter is located at that point.

We calculated the margin volume normalized to the margin volume for the COG method by varying the isocenter position to search the optimal value. Figure 3 shows an example of normalized margin volume distribution with *M*_S_ = 2 mm, rotational uncertainty is 0.5°. The distance between the two targets is 100 mm. The diameters (*D*1 and *D*2) of the two targets are 6 and 15 mm, respectively. The isocenters of the NSO and ASA are the same. The margin volume of the optimal isocenter is 6.8% less than that of the COG.

**FIGURE 4:**
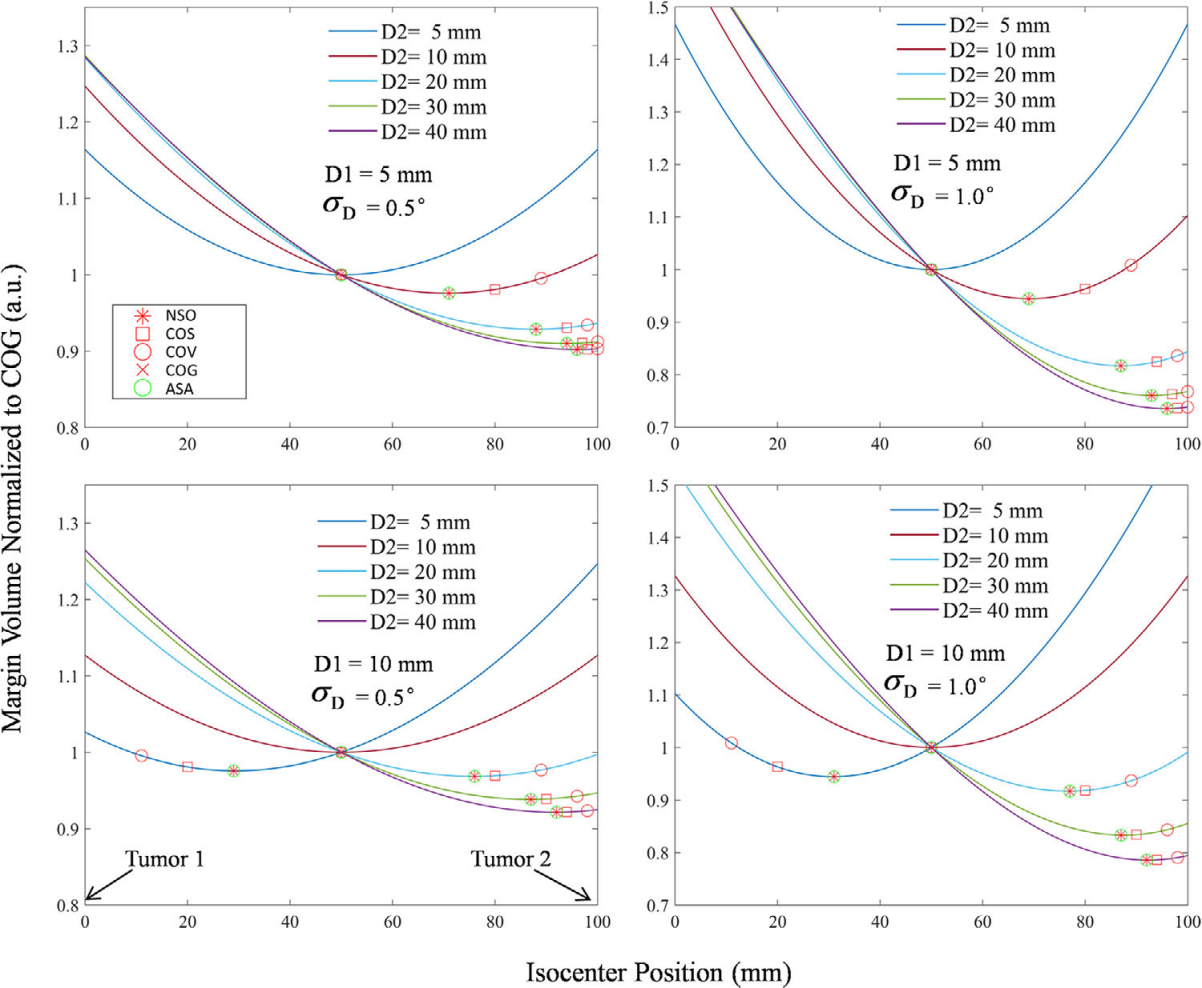
Margin volume normalized to *V*_COG_ as a function of isocenter position for various tumor diameters (*D*1, *D*2), *σ*_D_. The distance between the two tumors is 100 mm, and the isocenter position changes along the line between the two tumors. In the top two curves, the parameter sets are *D*1 = 5 mm, *M*_S_ = 2 mm, *σ*_D_ = 0.5° for left curve, and *σ*_D_ = 1.0°for right curve. In the bottom two curves, the parameter sets are *D*1 = 10 mm, *M*_S_ = 2 mm, *σ*_D_ = 0.5° for left curve, and *σ*_D_ = 1.0°for right curve. Center‐of‐geometric (COG) is located at 50 mm that is in the middle of the two tumors. The optimal isocenter position obtained by adaptive simulated annealing (ASA) is represented by a green hollow circle that is in the same position obtained by numerical simulation optimal (NSO) (red asterisk). The center‐of‐volume (COV) and center‐of‐surface (COS) are marked by red hollow circles and squares, respectively.

Figure 4 provides the margin volume normalized to COG as a function of isocenter position for various tumor diameters (*D*1, *D*2), *σ*_D_. Each curve plots the normalized margin volume versus isocenter position (changes along the line between the two tumors) with *D*2 was varied as 5, 10, 20, 30, and 40 mm.

From Figure 4, it is clear that the ASA method can find the optimal isocenter accurately. When the sizes of the two tumors are equal, the optimal isocenter obtained by ASA and numerical simulation coincides with COG, COV, and COS. When the size of two tumors is different, the optimal isocenter is close to the large tumor. Compared with the normalized margin volume at COG, the normalized margin volume at optimal point can be reduced by up to 9.8%, 7.8%, 26.4%, and 21.4% for *D*1 and *σ*_D_ of [*D*1 = 5 mm, *σ*_D_ = 0.5°], [*D*1 = 10 mm, *σ*_D_ = 0.5°], [*D*1 = 5 mm, *σ*_D_ = 1.0°], and [*D*1 = 10 mm, *σ*_D_ = 1.0°], respectively. The details about the normalized optimal margin volume of the curves in Figure 4 can be found in Table 1.

**TABLE 1:** The results of the optimal margin volume normalized to center‐of‐geometric (COG) with a series of *D*1, *D*2, and σ_D_

*σ*_D_	0.5°		1.0°	
*D*1	5 mm	10 mm	5 mm	10 mm
*D*2 = 5 mm	1	0.976	1	0.945
*D*2 = 10 mm	0.976	1	0.945	1
*D*2 = 20 mm	0.929	0.969	0.817	0.917
*D*2 = 30 mm	0.910	0.939	0.761	0.833
*D*2 = 40 mm	0.902	0.922	0.736	0.786

### 3.2 Multiple tumors

**FIGURE 5:**
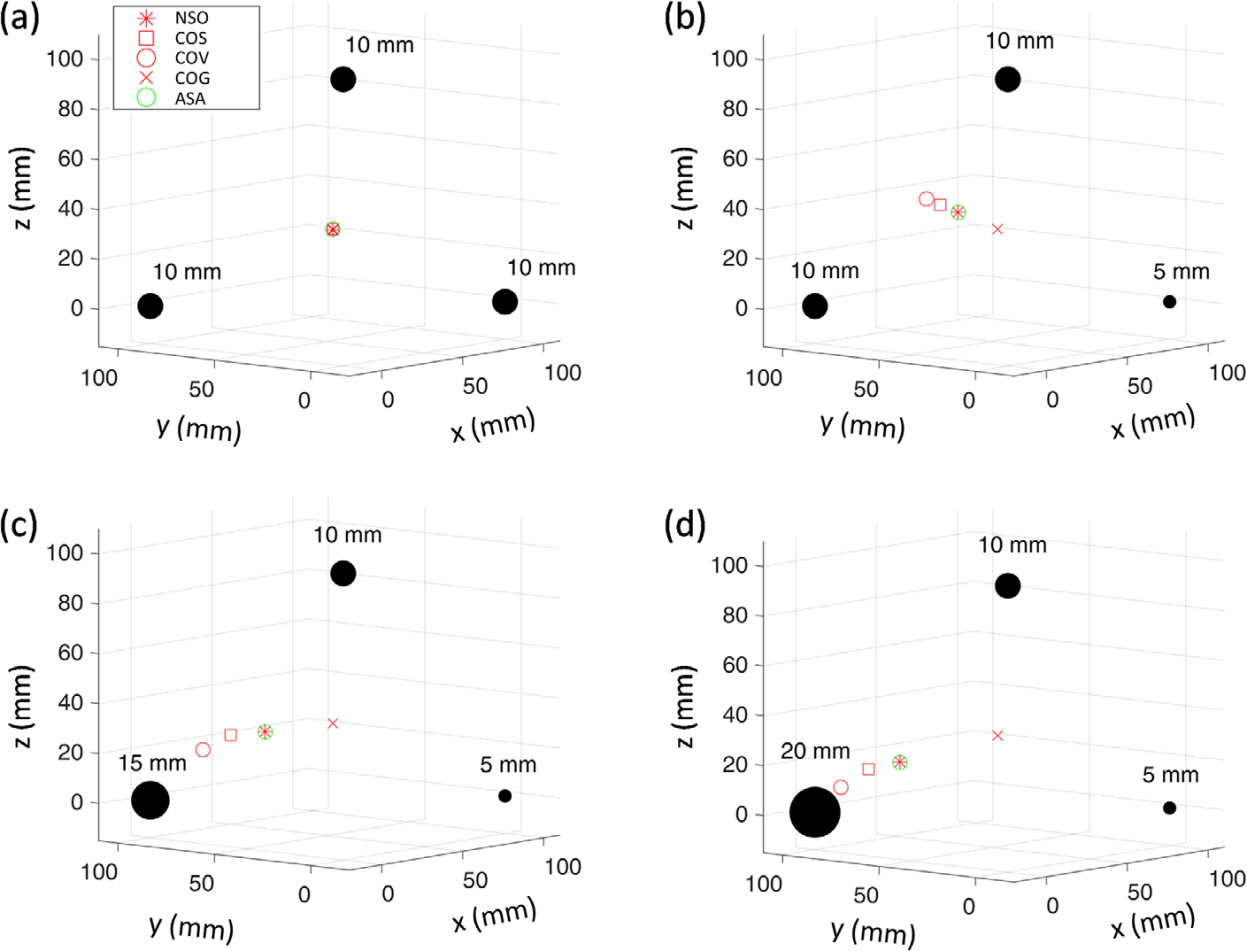
The positions of isocenter with numerical simulation optimal (NSO), center‐of‐surface (COS), center‐of‐volume (COV), center‐of‐geometric (COG), and adaptive simulated annealing (ASA) for three tumors. The diameters of tumors are (a) 10, 10, and 10 mm; (b) 5, 10, and 10 mm; (c) 5, 10, and 15 mm; and (d) 5, 10, and 20 mm, respectively.

The positions of optimal isocenter for three tumors were also calculated. As an example, Figure 5 shows several isocenter positions of NSO, COS, COV, COG, and ASA with *M*_S_ = 2 mm, *σ*_D_ = 1.0°. The coordinates of the center of three tumors are (100 mm, 0, 0), (0, 100 mm, 0), and (0, 0, 100 mm), respectively. During the numerical simulation, the number of the calculated points in the three‐dimensional space is 10.^6^

**FIGURE 6:**
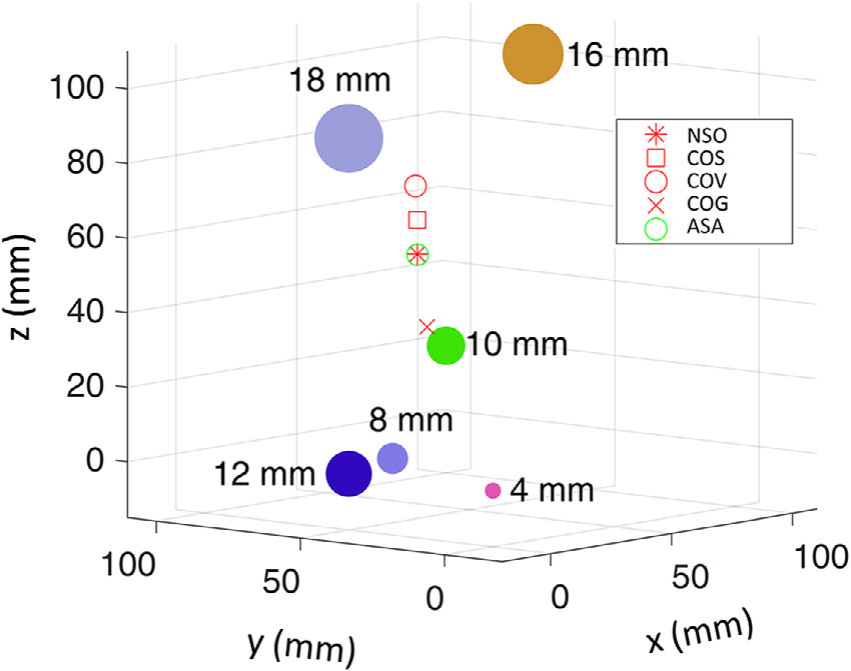
The positions of isocenter with numerical simulation optimal (NSO), center‐of‐surface (COS), center‐of‐volume (COV), center‐of‐geometric (COG), and adaptive simulated annealing (ASA) for six tumors. The diameters of tumors are 4, 8, 10, 12, 16, and 18 mm, respectively.

All the points will coincide when all the tumor sizes are the same. The optimal isocenter location will get close to the largest tumor when the tumor size increases. The volume at optimal point can be reduced by 2%, 6%, and 12% for the three different sizes compared to the volume of the COG method. The calculation times of numerical simulation and ASA are 1.8 h and 15 s, respectively. Figure 6 shows the positions of isocenter get by NSO, COS, COV, COG, and ASA for six tumors. The diameters of tumors are 4, 8, 10, 12, 16, and 18 mm, respectively. The calculation times of numerical simulation and ASA are 3.5 h and 19 s, respectively.

### 3.3 Clinical cases

Table 2 shows the tumor number, maximum dimensions, distances among tumors, the margin volume, and calculation time required of each case for multiple clinical brain tumors with a single isocenter placed at optimal point. The margin volume with isocenter at COG ranges from 6.3 to 22.3 cm^3^. Compared with the margin volume with isocenter at COG, the margin volume can be reduced by up to 27.7% (case 2) when the treatment center is at optimal point. Compared with the margin volume with isocenter at COS, the margin volume can be reduced by 0.1%–1.8% when the treatment center is at optimal point. Moreover, COS is a good approximation of optimal for clinical cases. The calculation time required for different tumors varies greatly obviously. For the plan with two tumors, the calculation time of ASA and numerical simulation is short (about 2 s). When the number of tumors exceeds two, the calculation time required for numerical simulation ranges from 18.8 min to 3.6 h. However, it only needs 5.0–19.8 s for ASA to find the optimal solution.

**TABLE 2:** The margin volume and calculation time required for multiple clinical brain tumors with a single isocenter placed at optimal point

						Time cost	
Case	Tumor number	Maximum dimension (mm)	Separation distances (mm)	*V*_COG_ (cm^3^)	(*V*_Optimal_ − *V*_COG_)/*V*_COG_ (%)	NSO	ASA (s)
1	2	1. 25.2 2. 18.1	*D* = 64.5	8.1	−1.5	1.6 s	1.8
2	2	1. 28.4 2. 4.8	*D* = 117.2	10.2	−27.7	2.1 s	1.9
3	3	1. 30.2 2. 7.9 3. 6.1	*D*_min_ = 62.4 *D*_max_ = 116.8	11.4	−15	20.4 m	5.4
4	5	1. 21.2 2. 8.5 3. 7.3 4. 10.2 5. 6.9	*D*_min_ = 46.8 *D*_max_ = 135.5	11.0	−5.7	2.2 h	17.1
5	4	1. 7.9 2. 29.5 3. 6.1 4. 8.2	*D*_min_ = 49.7 *D*_max_ = 128.4	18.9	−23.9	1.9 h	14.4
6	5	1. 14.8 2. 8.7 3. 7.5 4. 6.9 5. 18.6	*D*_min_ = 28.5 *D*_max_ = 72.8	9.2	−1.6	57.6 m	11.7
7	6	1. 8.2 2. 11.6 3. 7.9 4. 19.2 5. 5.4 6. 24.9	*D*_min_ = 39.4 *D*_max_ = 122.5	22.3	−4.2	3.6 h	19.8
8	3	1. 22.6 2. 9.7 3. 7.4	*D*_min_ = 62.5 *D*_max_ = 84.7	8.3	−10.1	18.8 m	5.0
9	4	1. 10.2 2. 8.6 3. 15.4 4. 7.8	*D*_min_ = 39.2 *D*_max_ = 68.5	6.3	−2.3	50.1 m	11.2
10	3	1. 12.7 2. 24.5 3. 9.8	*D*_min_ = 78.4 *D*_max_ = 102.5	12.2	−8.3	1.8 h	13.5

Abbreviations: ASA, adaptive simulated annealing; COG, center‐of‐geometric; NSO, numerical simulation optimal; *V*_COG_, margin volume with isocenter at COG; *V*_optimal_, margin volume with isocenter at optimal point.

## 4 DISCUSSION

We estimated a method to find the optimal isocenter position that minimizes the margin volume for multiple targets with brain SRS quickly. This method is based on a nonuniform‐margin expansion considering both the conventional translational error and the additional rotational error. This method searches the optimal isocenter position within the spatial range determined by multiple targets. To prove the advancement of this method, we calculated and compared the volume difference between *V*_optimal_ and *V*_COG_.

According to Equation (1), the expansion margin is proportional to the distance between the tumor boundary and the isocenter. When there are two tumors, the optimal isocenter position is on the line connecting the centers of two tumors. From Figures 3 and 4, it is observed that the optimal isocenter position is related with the size of two tumors and rotational uncertainty (*σ*_D_). When the two tumor sizes are the same size, the optimal point is located at the geometric center of the two tumors. When one tumor gradually becomes larger, the optimal point is getting closer and closer to the larger tumor. Moreover, the optimal isocenter position may enter the inside of the large tumor if the difference of two tumor sizes is large enough (e.g., *D*1 = 5 mm, *D*2 = 40 mm).

For a modern IGRT program equipped with kV onboard imaging device and 6° couch, the residual rotational uncertainty is generally about 0.5°–1.0°.^19^, ^20^, ^21^, ^22^ In this paper, the results of the optimal margin volume with *σ*_D_ = 0.5° and 1.0° were calculated. As shown in Table 1, the rotational uncertainty (*σ*_D_) has an important influence on the margin volume of optimal isocenter. When the rotational uncertainty (*σ*_D_) increases and other conditions are the same, a lower normalized margin volume for optimal isocenter can be obtained. The normalized margin volume at optimal point can be reduced by up to 26.4% when *D*1 = 5 mm, *D*2 = 40 mm, and *σ*_D_ = 1.0°. From the perspective of trend changes, more margin volume at optimal point may be reduced if *σ*_D_ becomes larger.

As shown in Figures 4, 5, 6, the ASA method can find the position of the optimal isocenter accurately. For each selected isocenter, it needs to calculate the nonuniform margin of each boundary point of all tumors. So the calculation amount of nonuniform margin is much larger than that of uniform margin. When performing numerical simulation, the time required for the calculation is closely related to the possible space range and resolution of the solution. For two tumors, the range of finding the solution is on a line, and the computational workload is relatively small. As shown in Table 2 (case 1 and case 2), the calculation time of numerical simulation and ASA is similar, which is clinically acceptable. For three or more tumors, the range of finding the solution is on a three‐dimensional space. The calculation time of numerical simulation would usually take from tens of minutes to several hours, which is clinically unacceptable. However, the ASA method generally only takes less than 20 s, which can well meet clinical needs.

In Figures 4, 5, 6, we have also marked the position of the COV and COS points. It can be seen that the COS point is closer to the optimal point than the COV point for all cases. This may be because the margin is more susceptible to the edge points of the tumor. In some cases (e.g., the sizes of the two tumors are quite different), the COS point is very close to the optimal point and the optimal point may be approximately replaced by the COS point. But this must be particularly careful, because the difference between the two points cannot be ignored under some conditions. For example, there will be 11 mm difference in isocenter position when *D*1 = 5 mm, *D*2 = 10 mm, *σ*_D_ = 1.0°, as shown in Figure 4.

The margin volume obtained by the nonuniform algorithm is related to the values *d* and *σ*_D_. When the values *d* and *σ*_D_ are small (e.g., *d* = 36 mm for *σ*_D_ = 0.45°), margin volume obtained by the nonuniform algorithm will differ slightly from that obtained by the uniform algorithm.^7^ Correspondingly, the margin volume will be less related to the location of the treatment isocenter. So this method is particularly suitable for the plans in which the tumors with quite different sizes are far apart.

The method was implemented in the Pinnacle^3^ treatment planning system. Pinnacle^3^ scripts, custom Python code, and ASA program written in standard C language were used to export tumor contour, extract tumor points, and find the optimal isocenter. By simply modifying the script that exports the tumor RT‐structure, this method can also be used in other treatment planning system (e.g., Eclipse or RayStation).

It should be noted that we focused on the relationship between a nonuniform‐margin volume with isocenter position in this study. Moreover, the influence of other factors on isocenter position is beyond the scope of this paper. But these factors (e.g., important organs at risk near tumors, collision during treatment delivery) should be considered when determining the final isocenter for clinical application. Further research may be carried out to investigate the relationship between these factors and the optimal isocenter position. For example, it might be feasible to consider incorporating planning organs‐at‐risk volumes into the optimization problem.

## 5 CONCLUSIONS

In this paper, we propose a method to select the isocenter position for multiple targets that minimizes the total margin volume. This method is based on a statistical model considering both the conventional translational error and the additional rotational error. In order to find the optimal solution quickly, an ASA algorithm was used in this study. The advantage of this method is that it will minimize the margin volume for given tumors to obtain same significance level. This method could be used for clinical isocenter selection and might be useful for the protection of normal tissue nearby.

## CONFLICT OF INTEREST

The authors declare no conflict of interests.

## AUTHOR CONTRIBUTIONS

Junjie Miao performed the simulation, analyzed the data, and wrote the manuscript. Yingjie Xu contributed to the data analyses and manuscript preparation. Jianrong Dai supervised the whole study. All authors have read and approved the manuscript.

## Data Availability

The data that support the findings of this study are available on request from the corresponding author.
